# Retroactive Maintains Cuticle Integrity by Promoting the Trafficking of Knickkopf into the Procuticle of *Tribolium castaneum*

**DOI:** 10.1371/journal.pgen.1003268

**Published:** 2013-01-31

**Authors:** Sujata S. Chaudhari, Yasuyuki Arakane, Charles A. Specht, Bernard Moussian, Karl J. Kramer, Subbaratnam Muthukrishnan, Richard W. Beeman

**Affiliations:** 1 Department of Biochemistry, Kansas State University, Manhattan, Kansas, United States of America; 2 Division of Plant Biotechnology, College of Agriculture and Life Science, Chonnam National University, Gwangju, Korea; 3 Department of Medicine, University of Massachusetts, Worcester, Massachusetts, United States of America; 4 Interfaculty Institute for Cell Biology, Animal Genetics, University of Tübingen, Tübingen, Germany; 5 Center for Grain and Animal Health Research, Agricultural Research Service, United States Department of Agriculture, Manhattan, Kansas, United States of America; Howard Hughes Medical Institute, United States of America

## Abstract

Molting, or the replacement of the old exoskeleton with a new cuticle, is a complex developmental process that all insects must undergo to allow unhindered growth and development. Prior to each molt, the developing new cuticle must resist the actions of potent chitinolytic enzymes that degrade the overlying old cuticle. We recently disproved the classical dogma that a physical barrier prevents chitinases from accessing the new cuticle and showed that the chitin-binding protein Knickkopf (Knk) protects the new cuticle from degradation. Here we demonstrate that, in *Tribolium castaneum*, the protein Retroactive (TcRtv) is an essential mediator of this protective effect of Knk. TcRtv localizes within epidermal cells and specifically confers protection to the new cuticle against chitinases by facilitating the trafficking of TcKnk into the procuticle. Down-regulation of TcRtv resulted in entrapment of TcKnk within the epidermal cells and caused molting defects and lethality in all stages of insect growth, consistent with the loss of TcKnk function. Given the ubiquity of Rtv and Knk orthologs in arthropods, we propose that this mechanism of new cuticle protection is conserved throughout the phylum.

## Author Summary

The outer shell of an insect serves both as protective skin and rigid exoskeleton that must be periodically replaced with a new, larger one during development. During this molting process, the inner layers of the old exoskeleton are digested and recycled, while the outer layers are discarded. Secretion of the new skin necessarily commences before the partial recycling and shedding of the old shell. This creates a problem for the insect, namely how to protect the new skin from digestive enzymes intended for the old shell that closely enwraps it. Previously we showed that such protection is afforded by the Knickkopf (Knk) protein, which is secreted from the epidermis and infiltrates the new skin, rendering it resistant to enzymatic degradation. In this work, we show that another protein, called Retroactive (Rtv), ensures the proper trafficking of Knk into the newly secreted skin. Rtv remains inside the epidermal cells, while directing the transport of Knk to the cell surface and ensuring its export into the new skin. Digestive enzymes are then secreted and target the old exoskeleton while leaving the new one intact. This dependence of Knk on Rtv function is probably true for all insects and other arthropods.

## Introduction

A critical feature of the insect molting process is the simultaneous synthesis and degradation of chitin at different sites associated with the cuticle [1], [2]. Chitinolytic enzymes (molting fluid chitinases and N-acetylglucosaminidases) dissolve parts of the old chitinous exoskeleton into oligomeric and monomeric N-acetylglucosamine (GlcNAc) units, which are subsequently recycled into the collective pool of biosynthetically derived activated precursors of GlcNAc monomers (UDP-GlcNAc) used for the synthesis of new cuticular chitin [3], [4]. The enzyme that catalyzes the addition of these monomers onto the growing chitin oligosaccharide is the membrane-bound chitin synthase [3], [5], [6]. Upon synthesis and secretion into the extracellular (subcuticular) space by epidermal cells, nascent chitin aggregates into microfibrils and accumulates within the assembly zone (the layer immediately above the apical surface of the epidermal cells) to eventually organize into new cuticular laminae [7], [8].

At the ultrastructural level, the new cuticle extends from its inner boundary on the apical side of the epidermal cell membrane to its outer “envelope” layer that appears to partition it from the overlying old procuticle [9]. However the envelope layer functions only as a structural boundary at the early stages of molting, and does not function as a barrier against molting fluid chitinases [10]. Indeed, the molting fluid chitinase of *T. castaneum* (TcCht-5) was observed throughout the new procuticle co-localized with chitin. The mysterious stability of the new cuticle in intimate contact with enzymatically active chitinases was shown to be due to its physical association with the cuticle assembly protein, Knickkopf (TcKnk; homologue of *D. melanogaster* knickkopf, DmKnk) [10].

Like DmKnk, the protein Retroactive from *Drosophila melanogaster* (DmRtv) was also previously demonstrated to affect cuticle differentiation and tracheal tube morphogenesis [11], [12], [13]. DmRtv was predicted to form a membrane-anchored, three-finger loop structure that interacts with chitin via two aromatic residues situated in each loop [11]. Although a direct role for this protein in cuticular chitin filament organization in embryonic tracheal tubules of *D. melanogaster* was envisaged, the mechanism of this ordering process is unclear. In the current study we show that a direct effect of the *T. castaneum* Rtv homologue (TcRtv) on cuticular chitin organization is unlikely, given its predominant intracellular distribution within epidermal cells. Instead, its activity is shown to be crucial for the proper trafficking of TcKnk to the procuticle where the latter protein associates with and protects chitin. Hence, TcRtv promotes cuticle synthesis and organization by facilitating TcKnk's localization in the integument.

## Results

### TcRtv is an evolutionarily conserved protein required for insect molting

**Figure 1:**
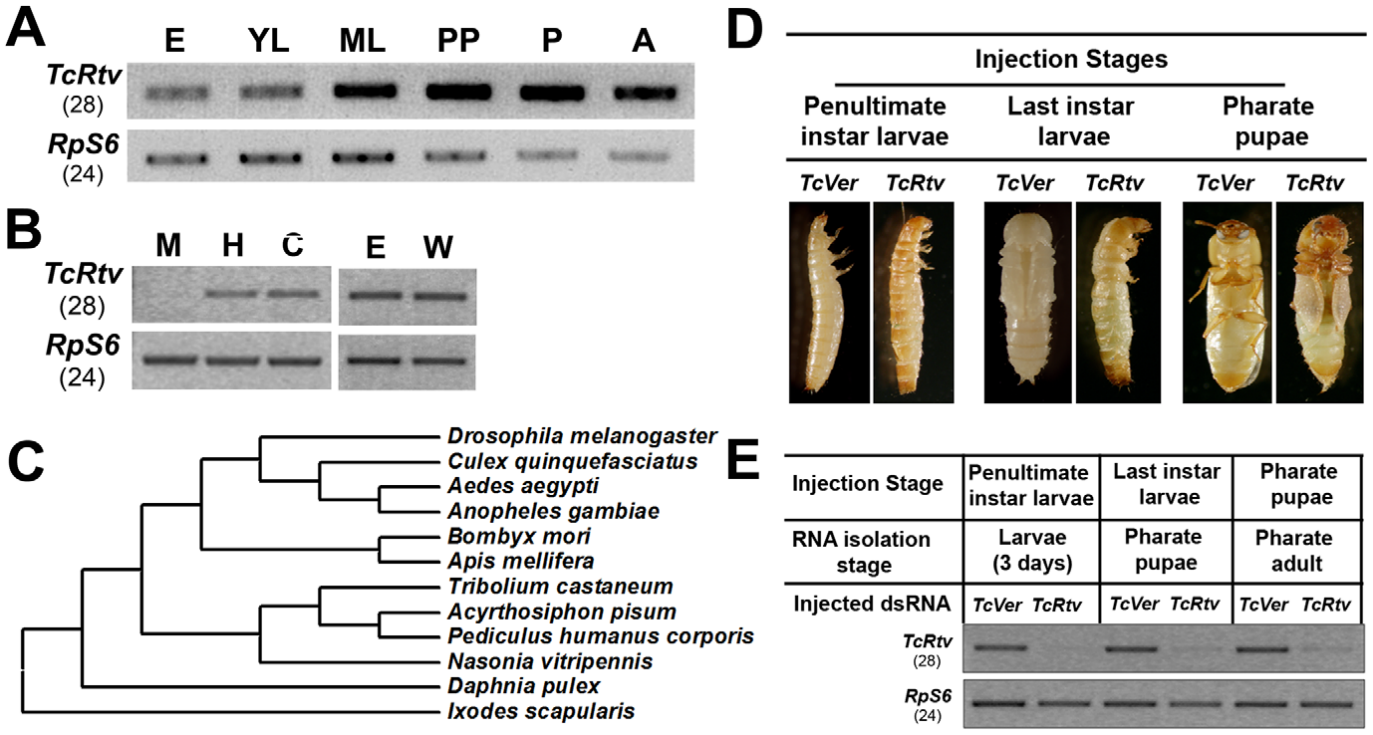
TcRtv is a conserved protein required for insect molting. (A) Expression of *TcRtv* during *T. castaneum* development. Total RNA was extracted at different stages of development: E, eggs; YL, young larvae; ML, mature larvae (last instar larvae); PP, pharate pupae; P, pupae and A, adult. RT-PCR (28 cycles) was performed using cDNA prepared from total RNA and gene-specific primers. *TcRpS6* (*T. castaneum ribosomal protein-S6*) was used as an internal loading control (24 cycles). (B) Tissue-specific expression pattern of *TcRtv* in *T. castaneum* late last instar larval midgut (M), hindgut (H) and carcass (C) (whole body minus gut) and pharate pupal elytra (E) and hindwing (W) was detected by RT-PCR using gene specific primers (28 cycles). *TcRpS6* was used as an internal loading control (24 cycles). (C) Phylogenetic analysis of Rtv orthologs from several orders of insect species was done using MEGA 4.0 neighbor joining method, with *Ixodes scapularis* included as outlier. (D) Effect of *TcRtv* dsRNA treatment on development of *T. castaneum* (n = 20). dsRNA for the *Vermilion* gene (*TcVer*) was used as a control (n = 20 each). (E) Effect of *TcRtv* dsRNA treatment on the transcript levels. RT-PCR was performed (28 cycles) to check the level of *TcRtv* transcripts using gene-specific primers. *TcRpS6* was used as a loading control (24 cycles).

TBLASTN and BLASTP searches of the *T. castaneum* genome using the DmRtv protein sequence as query identified only a single gene coding for an orthologous protein. *T. castaneum retroactive* (*TcRtv*) (JX470185) maps on LG4, position 24.3 cM. *TcRtv* is composed of three exons and encodes a 150- amino acid residue long protein with a predicted C-terminal GPI anchor and an ω-asparagine at position 124 (Figure S1A and S1B) (GPIPred). A predicted cleavable signal peptide at its N-terminus suggests that this protein enters the ER secretory pathway prior to GPI-anchoring and is transported to the cell surface via intracellular secretory vesicles. Expression analysis revealed the presence of *TcRtv* transcripts throughout development including embryonic, larval, pupal and adult stages (Figure 1A). Lower levels of *TcRtv* expression were detected at the embryonic and young larval stages relative to later stages of development. Tissue-specific expression of *TcRtv* revealed that *TcRtv* transcripts could only be detected in the hindgut and carcass but not in midgut tissues indicating that this gene is expressed in tissues of ectodermal origin (Figure 1B). *TcRtv* transcripts were also detected in pharate adult elytra and hindwings (Figure 1B).

When other fully sequenced arthropod genomes were queried with DmRtv or TcRtv sequences, only a single homolog was identified in all the insect species. The presence of an ortholog of *TcRtv* gene in all sequenced insect genomes suggests an essential function for this protein in insect survival and development (Figure 1C). Interestingly, a single *Rtv* ortholog is also present even in non-insect arthropod species including the water flea, *Daphnia pulex*, and the deer tick, *Ixodes scapularis*, suggesting that *Rtv* may have a conserved biological function in all species that produce a chitinous exoskeleton. On the other hand, we could not identify *Rtv* homologs in the genome of the nematode, *Caenorhabditis elegans*, which does synthesize chitin in its eggshells and the pharyngeal lumenal walls. Thus Rtv may have appeared early during arthropod evolution.

Given the ubiquitous presence of *TcRtv* transcripts during insect growth, we hypothesized a critical role for this protein in post-embryonic development. To test this hypothesis, we depleted *TcRtv* transcripts via RNA interference (RNAi) by administration of *TcRtv*-specific dsRNA (*dsRtv*) during multiple developmental stages of *T. castaneum*. RNAi of *TcRtv* led to molting arrest at larval-larval, larval-pupal or pupal-adult stages, depending upon the developmental stage at the time of injection, with significant reductions in transcript levels at each stage tested (Figure 1D and 1E). Apolysis and partial slippage of the old larval cuticle proceeded at the subsequent molt after injection of *TcRtv* dsRNA into penultimate-instar larvae, but the larvae failed to complete the larval-larval molt and remained entrapped within their old larval cuticle. Similarly, insects injected with *TcRtv* dsRNA at later stages were arrested at the larval-pupal or pupal-adult molts and failed to shed their old cuticle. These phenotypes were reminiscent of those that we have reported previously following RNAi for *TcChs-A* [5]. Control insects injected with the same dose of dsRNA for the eye pigmentation gene, *TcVer* (*T. castaneum Vermilion*), developed normally except for the loss of eye color. These results demonstrate that Rtv is essential for molting in *T. castaneum*.

### TcRtv is important for the maintenance of procuticular chitin

**Figure 2:**
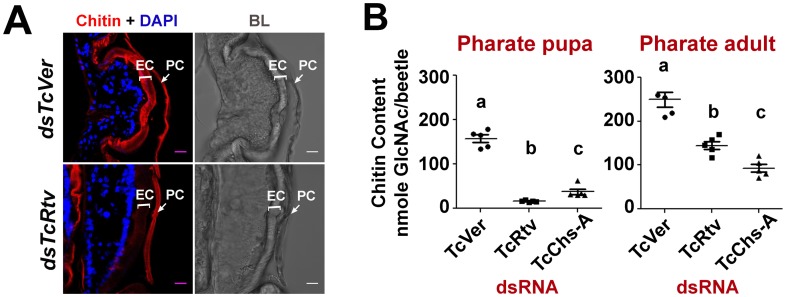
TcRtv is important for the sustenance of procuticular chitin. (A) Chitin staining (red) of cryosections of pharate adult insects containing both the old pupal cuticle (PC) and the newly synthesized elytral cuticle (EC). Nuclei were stained with DAPI (blue). Scale bar, 5 µm. (B) Quantitative analysis of chitin content from whole animals at pharate pupal and pharate adult stages was carried out using a modified Morgan-Elson assay (n = 5). *TcVer* and *TcChs-A* dsRNA-treated insects were used as controls. *TcRtv* RNAi resulted in a significant loss of chitin in comparison with the *TcChs-A* knockout. Data are reported as mean ± SE (n = 5 each). Statistical significance was computed with Student's t test. Means identified by different letters (a, b and c) are significantly different at P<0.05.

**Figure 3:**
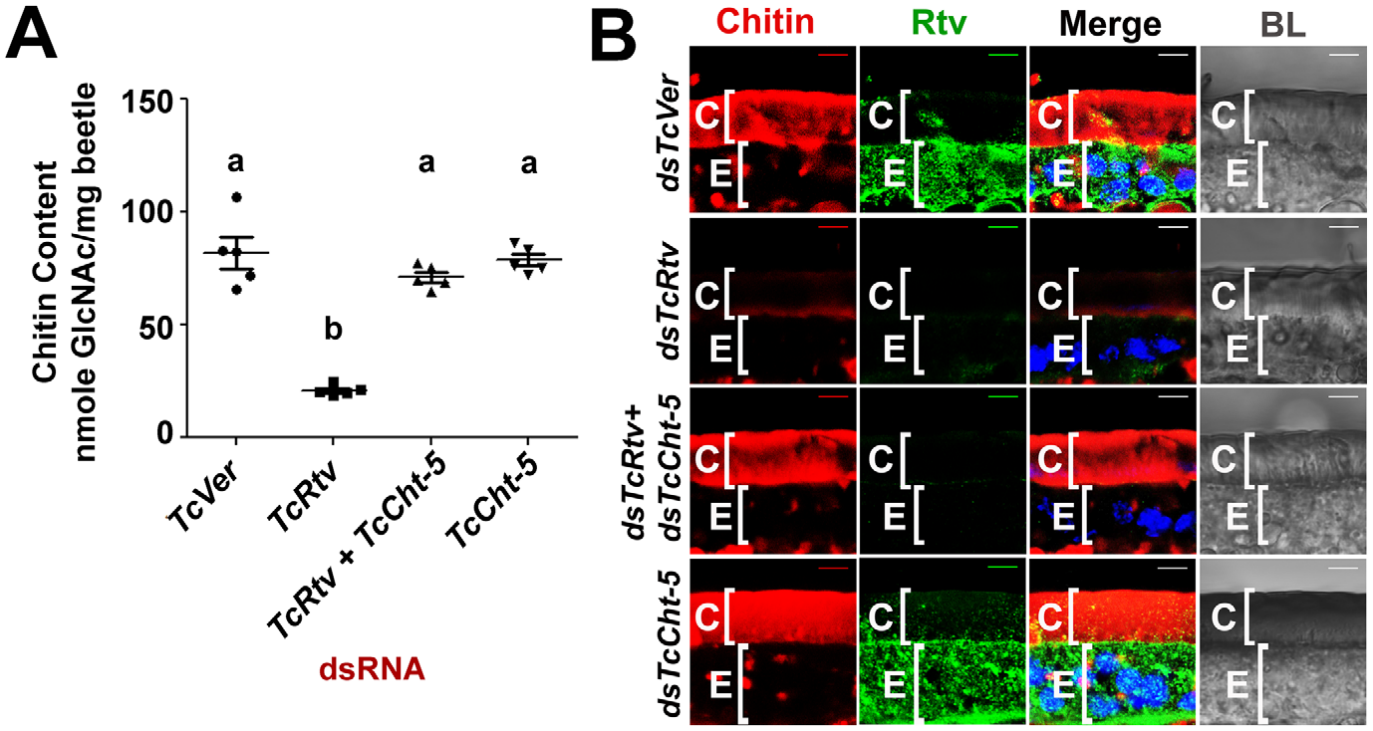
TcRtv prevents chitinase-mediated degradation of procuticular chitin. (A) Quantitative analysis of total chitin content of pharate adult insects after dsRNA treatment for *TcVer*, *TcRtv*, *TcRtv*+*TcCht-5* and *TcCht-5* was performed using a modified Morgan-Elson assay (n = 5). Chitin depletion resulting from *TcRtv* RNAi was rescued following double RNAi for both *TcRtv* and *TcCht-5*. Data are reported as mean ± SE (n = 5 each). Statistical significance was computed with Student's t-test. Means identified by different letters (a and b) are significantly different at P<0.05. (B) Immunohistochemical analysis of *TcVer*, *TcRtv*, *TcRtv*+*TcCht-5* and *TcCht-5* dsRNA-treated insects revealed rescue of the chitin level upon co-knockdown of *TcRtv* and *TcCht5* (*dsTcRtv+dsTcCht-5*). Chitin (red), TcRtv (green), DAPI (blue), C, cuticle; E, epithelial cell. Scale bar = 5 µm.

Because of the terminal phenotypic resemblance of insects treated with dsRNAs against *TcRtv* or *TcChs-A* [5], we suspected a reduction of procuticular chitin content resulting in loss of mechanical strength of the new cuticle may account for the failure to complete ecdysis. To test this hypothesis, insects at the pharate pupal stage were treated with *TcRtv*-specific dsRNA and their chitin contents were probed qualitatively and quantitatively just prior to the adult molt (pharate adult). Confocal microscopic analysis of the pharate adult elytra stained with a chitin-binding domain (CBD) probe (rhodamine-conjugated CBD) showed a near complete loss of chitin in the new cuticle of TcRtv-depleted insects when compared to control insects (Figure 2A). A similar reduction in new cuticular chitin was also detected in the pharate adult insect body wall after RNAi for *TcRtv* compared to *TcVer*-depleted insects (Figure 3B, column 1- row 2). Independently, quantitative analysis of total body chitin content by a modified Morgan-Elson assay confirmed chitin depletion in these insects during both larval-pupal and pupal-adult molts (Figure 2B). The reduction of chitin content following RNAi of *TcRtv* was comparable to that observed in TcChs-A-depleted insects, and varied depending on the stage of insect development from ∼2- to 10-fold relative to control insects (Figure 2B). Collectively, these data reveal that TcRtv affects molting by modulating the level of chitin, predominantly in the newly forming cuticle.

### TcRtv prevents chitinase-mediated degradation of procuticular chitin

The total amount of chitin in an insect is likely to change dynamically during periods of growth as a result of repeated cycles of cuticle deposition and turnover. The dynamics are more complex during molting when there is an overlap of the period of chitin synthesis in the new cuticle with that of chitin degradation in the old cuticle. As a result, several possible mechanisms by which TcRtv may regulate chitin levels in the procuticle can be envisioned. The loss of chitin following RNAi for *TcRtv* suggests that this protein might be an activator of chitin synthesis or an inhibitor of chitin degradation. In the first scenario, TcRtv might affect chitin synthesis via its effect on *TcChs-A* at the transcriptional, post-transcriptional, translational or post-translational levels. However, our observation that steady-state levels and cellular distribution of TcChs-A protein were similar in control and TcRtv-depleted insects (Figure S2) argues against a direct effect of TcRtv on TcChs-A expression and localization. An alternative hypothesis that would explain the observed reduction in chitin in the newly forming procuticle following *TcRtv* RNAi is that TcRtv protects newly synthesized chitin from molting fluid chitinases. Indeed, we have shown recently that chitin is co-localized with chitinases, which are not excluded from the newly forming procuticle as had been assumed in the past. We have further shown that the chitin-binding protein, TcKnk, is needed to protect the nascent chitin in the procuticle from chitinase-mediated degradation [10]. To test the hypothesis that TcRtv also protects chitin, we down-regulated the expression of the major molting fluid chitinase (*TcCht-5*) at the pharate adult stage either alone or in combination with *TcRtv* and subjected these samples to quantitative chitin content analysis. Remarkably, the analysis revealed that insects subjected to RNAi for *TcRtv* alone exhibited significant chitin reduction, but co-depletion of both *TcRtv* and *TcCht-5* transcripts resulted in nearly a complete rescue of cuticular chitin content relative to control levels (Figure 3A). These results also rule out a requirement for TcRtv for the synthesis and transport of chitin to the procuticle by Chs-A and suggest that Rtv-mediated stabilization of chitin involves a step after the synthesis of chitin.

This conclusion was further supported by confocal microscopy, which showed a near complete loss of chitin in the procuticle following depletion of *TcRtv* transcripts compared to the dsRNA *TcVer*-treated insects. But RNAi for both *TcCht-5* and *TcRtv*, rhodamine-CBD staining indicated that the level of chitin within the new cuticle of body wall was restored to the same level as *dsTcVer* controls (Figure 3B, compare row 3 with row 1).

### TcRtv affects the ultrastructure of the procuticle

**Figure 4:**
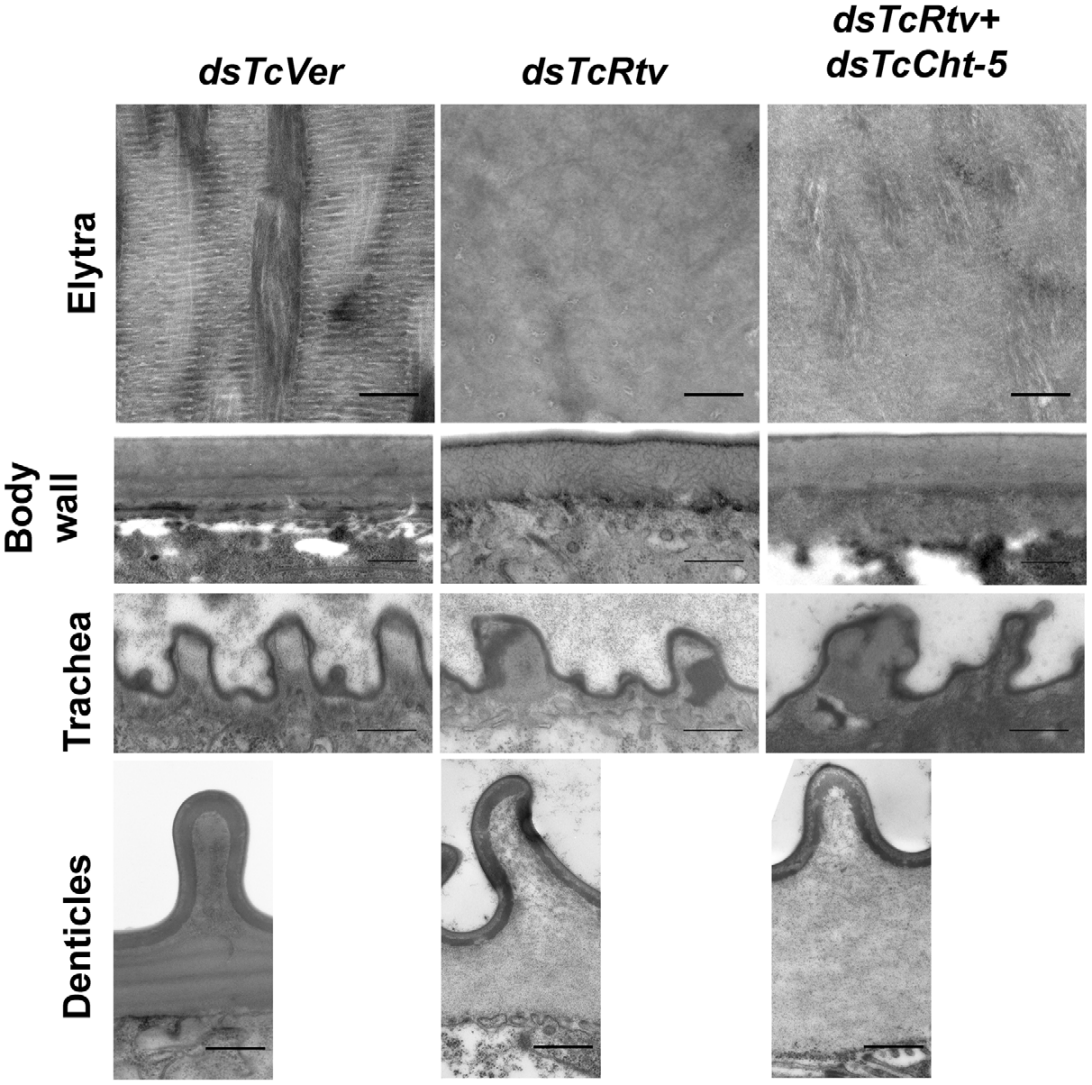
TcRtv is essential for maintenance of normal cuticle architecture. Transmission electron microscopic analyses of pharate adult elytral and dorsal body wall cuticles show loss of laminar organization upon *TcRtv* dsRNA (*dsTcRtv*) treatment (upper two panels). Although simultaneous down-regulation of *TcRtv* and *TcCht-5* transcripts resulted in the restoration of chitin within the procuticle, the laminar organization of chitin was not restored (panels marked *dsTcRtv+dsTcCht5*). dsRNA for *Vermilion* (*dsTcVer*) was used as a control. Compared to control, after RNAi for *TcRtv*, the tracheae were misshapen and had electron dense material. This phenotype was not restored to normal after simultaneous down-regulation of *Rtv* and *Cht5* genes (panels marked *dsTcRtv+dsTcCht5*). The panels in the bottom row show that the horizontal laminae were missing under the misshapen denticles after *TcRtv* dsRNA treatment. Co-injection of dsRNAs for *Rtv* and *Cht5* did not restore the normal phenotype. Scale bar = 500 nm.

To gain a better understanding of the nature of the restored chitin in the procuticle following depletion of transcripts for both *TcCht-5* and *TcRtv*, we performed Transmission Electron Microscopic (TEM) analysis of the elytra, body wall, tracheae and denticles of insects treated with the indicated combination of dsRNAs. Ultrathin sections of elytral and body wall cuticle from control insects were characterized by a well-organized laminar architecture. Along with the drastic reduction in procuticular chitin, the laminar architecture was completely disrupted following RNAi for *TcRtv*. Although simultaneous down-regulation of *TcRtv* and *TcCht-5* transcripts resulted in the restoration of chitin within the procuticle, the laminar organization was not reestablished (Figure 4). Interestingly, this phenotype was indistinguishable from the phenotype that we have recently observed in elytra of pharate adults depleted of transcripts for both *TcKnk* and *TcCht-5*.

Additional analyses also revealed structural deformities of denticles protruding at the lateral body wall that is consistent with loss of laminar architecture in denticular procuticle following down-regulation of *TcRtv* in these insects (Figure 4). Similarly the taenidial cuticle that lines the tracheal lumen and stabilizes the tracheal tube in these insects also appeared to exhibit structural defects possibly due to the disorganized procuticle (Figure 4) and electron-dense inclusions they harbored. Simultaneous down-regulation of *TcRtv* and *TcCht-5* transcripts, which restores chitin back to normal levels, did not rescue these phenotypes in either the denticles or taenidia, indicating the importance of TcRtv in maintaining the morphology of both taenidia and denticles (Figure 4).

### TcRtv is essential for the procuticular localization of TcKnk

**Figure 5:**
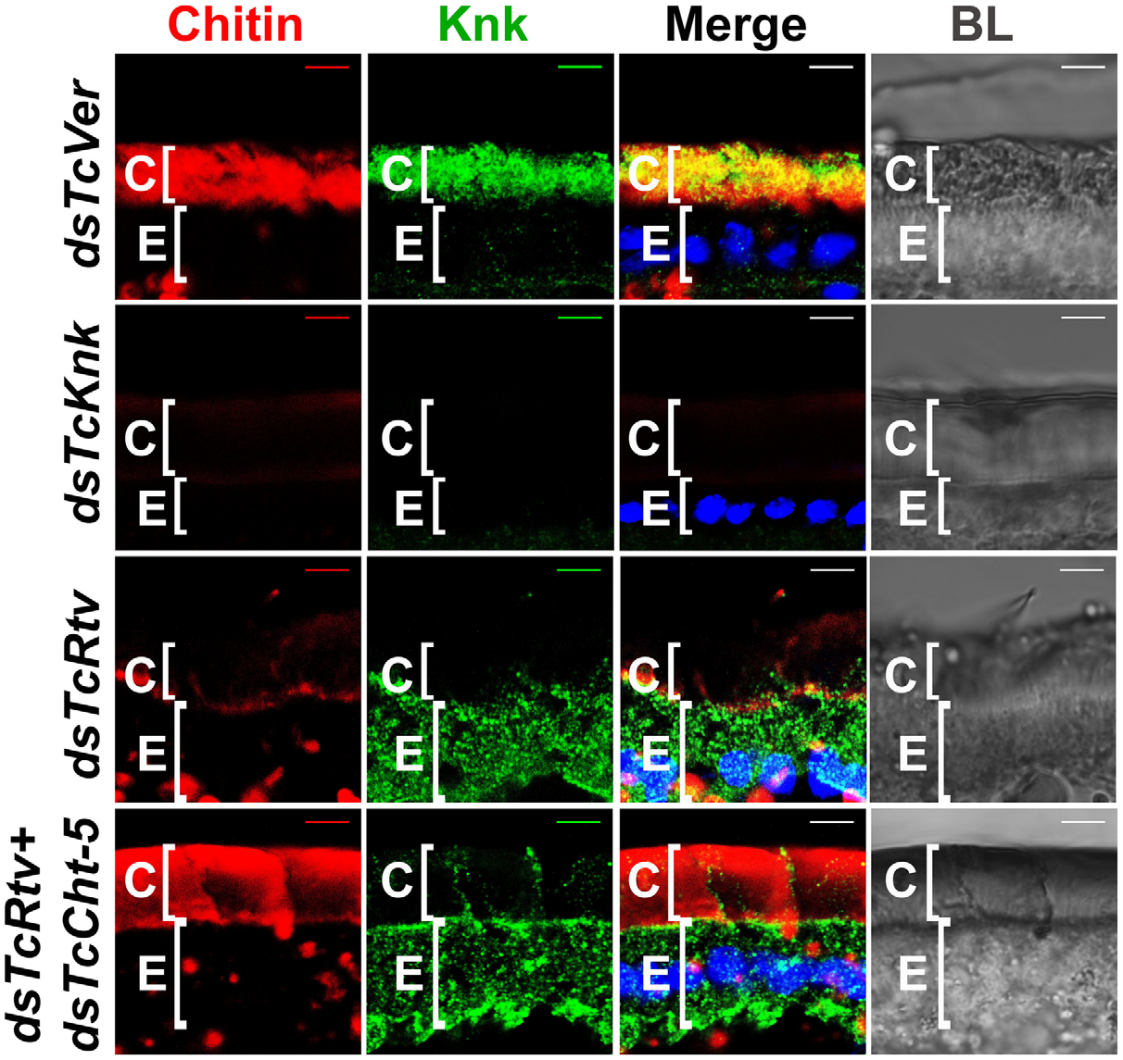
TcRtv influences the localization of TcKnk to the procuticle. Cryosections of *T. castaneum* pharate adult lateral body wall (20 µm thick) of control *TcVer* (*dsTcVer*), *TcKnk* (*dsTcKnk*), *TcRtv* (*dsTcRtv*) or *TcRtv+TcCht-5* (*dsTcRtv+dsTcCht-5*) dsRNA-treated insects were immunostained with *D. melanogaster* Knk (DmKnk) antiserum (green). Confocal microscopy reveals that Knk is localized predominantly in the procuticle (row 1). Mislocalization of TcKnk in pharate adults injected with *TcRtv* dsRNA (row 3) and co-injected with dsRNAs for *TcRtv* and *TcCht-5* is evident (row 4). Although chitin levels were restored to near normal levels in the procuticle after co-injecting dsRNA for *TcRtv* and *TcCht-5*, TcKnk was mislocalized, remaining entirely inside the cell rather than being secreted into the procuticle (row 4). Chitin (red), TcKnk (green), DAPI (blue), C, cuticle; E, epithelial cell. Scale bar = 5 µm.

Since the phenotypes of RNAi for *TcRtv* and *TcKnk* are indistinguishable, we considered the possibility that these two proteins may be involved in the same linear pathway. Immunohistochemistry carried out on sections of control insects (*dsTcVer*-treated) and those treated with dsRNAs specific for *TcRtv*, using a polyclonal antibody against Knk indicated that depletion of *TcRtv* transcripts alone resulted in the mislocalization of TcKnk from its normal distribution predominantly in the procuticle to an exclusive presence within the epidermal cells, perhaps with some TcKnk in the plasma membrane (Figure 5, compare row 3 with row 1).

However it is likely that the altered localization of TcKnk may have been brought about indirectly by a reduction of chitin which acts as a signal for TcKnk's release into the procuticle and not by a direct effect of TcRtv on TcKnk trafficking. To distinguish between these two possibilities, TcKnk localization was examined in insects depleted of both *TcRtv* and *TcCht-5* transcripts by co-injection of both dsRNAs. This enabled us to disable TcRtv function without concomitant depletion of procuticular chitin, and thus observe the direct effect of TcRtv on localization of TcKnk independently of any possible contributing effects of procuticular chitin itself. Although chitin levels remained at near normal levels in the procuticle after such treatment, TcKnk was mislocalized, remaining entirely inside the cell rather than being secreted into the procuticle (Figure 5, compare row 4 with row 1). The distribution of TcKnk in these double-knockout insects was indistinguishable from that observed in those treated with dsRNA for *TcRtv* alone. These observations support the hypothesis that TcRtv facilitates the trafficking of TcKnk into the procuticle.

## Discussion

The assembly of chitin into the laminated procuticle of insects is a complex process that involves the coordinated action of multiple proteins involved in the synthesis and modification of chitin as well as other chitin-binding and chitin-organizing proteins. While the functions of chitin synthases, deacetylases and chitinases have been studied in some detail [5], [14], [15], [16], the role of non-enzymatic chitin-binding and organizing proteins are less well understood [10], [17]. Mutational analysis in *D. melanogaster* has implicated two genes, *Knk* and *Rtv*, in the organization of tracheal tube chitin and of the embryonic cuticular matrix [11], [12]. More recently, we have shown that the TcKnk protein is localized primarily in the procuticle, possibly bound to chitin [10]. Furthermore, we have shown that procuticular TcKnk is crucial to the protection of chitin from the action of chitinases, which are not excluded from chitin in the newly forming procuticle as previously believed. In addition, TcKnk is needed for the organization of procuticular chitin into an ordered laminar array. However, the role of the protein TcRtv has not been studied extensively even though the phenotypes of *Rtv* and *Knk* mutants of *D. melanogaster* are virtually indistinguishable [11], [12].

Both TcKnk and TcRtv are predicted to encode proteins with GPI anchors by bioinformatic prediction tools (big-PI Predictor, PredGPI and GPI-SOM). However, experimental evidence for the GPI anchor is available only in the case of Knk. TcKnk and DmKnk are released from the plasma membrane of cells expressing this protein by treatment with phosphatidylinositol-specific phospholipase C (PI-PLC), an enzyme known to cleave the GPI anchor of membrane-bound proteins [10], [12]. TcRtv expressed in insect cell lines infected with a recombinant virus containing the Rtv coding sequences is not released by PI-PLC treatment (Figure S3). We suspect that TcRtv does not have a true GPI anchor and may merely have a membrane-spanning segment at the C-terminus, suggesting that this protein remains embedded in the ER or attached within vesicles and plasma membranes. Therefore, we propose that TcRtv may not have a direct role in protecting chitin from chitinases and that the loss of procuticular chitin observed after RNAi of *TcRtv* expression must be via an indirect effect. The finding that there are no significant differences in the amount or localization of TcChs-A in control and dsRNA *TcRtv*-treated insects also suggests that TcRtv does not have a direct effect on *TcChs-A* expression or localization. Finally, the full recovery of chitin levels in the procuticle in the absence of TcRtv upon down-regulation of chitinases rules out any role for TcRtv in the synthesis and transport of chitin to the procuticle.

Then how does TcRtv affect cuticle integrity? We have demonstrated that the absence of chitin in the procuticle alone does not account for the mislocalization of the TcKnk protein after RNAi-mediated transcript depletion of *TcRtv*, because simultaneous RNAi of *TcRtv* and *chitinase* genes, while restoring procuticular chitin, fails to rescue the intracellular mislocalization of TcKnk. Rather, it appears that the targeting of TcKnk protein to the procuticle requires the presence of TcRtv. Whether this is due to a direct protein-protein interaction between TcRtv and TcKnk or through a more complex targeting pathway remains to be established.

A search of the SCOP database revealed that the insect Retroactive proteins share broad structural similarity with the snake toxin-like superfamily of proteins with the three-finger domain (TFD) or Ly-6/uPAR domain implicated in protein-protein interactions (Figure S4) [11], [18]. The Ly-6 protein family is composed of several membrane-bound receptors, GPI-anchored proteins, and soluble ligands. These proteins are characterized by the presence of an approximately 100-residue long extracellular motif called the three-finger domain (TFD) [18]. In the prototypical structure described for proteins of this family, the sea snake venom neurotoxin or erabutoxin b, 10 cysteines form a series of disulfide bridges that stabilize the protein core and allow three finger-like loops to protrude [18], [19]. These finger-like extensions with variable sequences are believed to enable interaction of these proteins with a broad range of substrates or ligands, each with a high degree of specificity. Insect Rtv proteins contain 10 cysteines that are predicted to adopt the TFD fold (Figure S4) [11], suggesting that they may be members of the snake toxin-like superfamily of proteins.

Our work has uncovered a novel mechanism involved in cuticular chitin maintenance, wherein presence of TcRtv inside the cells triggers the accretion of TcKnk to the growing chitinous matrix of the procuticle whereupon the latter protein facilitates chitin organization and confers protection from chitinolytic enzymes during the process of molting [10]. The mechanism described herein is probably conserved in all chitinous invertebrates.

## Materials and Methods

### Insect cultures

The GA-1 strain of *T. castaneum* was used for all experiments. Insects were reared at 30°C in wheat flour containing 5% brewer's yeast under standard conditions as described previously [20].

### Identification of the *TcRtv* gene in the *T. castaneum* genome database

An extensive, genome-wide search for homologs of *DmRtv* in the *T. castaneum* genome database was performed using NCBI programs TBLASTN and BLASTP and using the amino acid sequence of DmRTV as query.

### Cloning and sequencing of a *TcRtv* cDNA

A DNA fragment containing the complete coding sequence of *TcRtv* (453 bp) was amplified by reverse transcriptase-PCR (RT-PCR) using the gene-specific primers (forward primer 5′- ATGGGTCTGTTTAGATCAATTT-3′ and the reverse primer 5′- TTACAAAAATCGTAAAATCAGTCT-3′) using cDNA prepared from the RNA extracted from different stages of beetle development as template. Additional 5′ and 3′ UTR sequences were obtained by 5′- and 3′- RACE. The amplified fragment was cloned into the pGEMT vector. Sequencing of the cDNA clone was carried out at the DNA sequencing facility at Kansas State University.

### Phylogenetic analysis of TcRtv

Rtv-like proteins were identified by TBLASTN searches of the fully sequenced genomes of *T. castaneum, Anopheles gambiae, Aedes aegypti, Culex quinquefasciatus, Bombyx mori, Acyrthosiphon pisum, Nasonia vitripennis* and *Ixodes scapularis* using the amino acid sequence of DmRtv as query. Multiple sequence alignments of TcRtv and the related Rtv-like proteins from other insects and arthropods were carried out using the ClustalW software prior to phylogenetic analysis. A consensus phylogenetic tree was constructed using MEGA 4.0 neighbor joining method [21].

### Determination of expression profiles of *TcRtv*

The isolation of RNA and preparation of cDNA from different stages of *T. castaneum* development were carried out as described previously [10]. These cDNA templates and the pair of *TcRtv*-specific primers, forward primer 5′-ATGGGTCTGTTTAGATCAATTT-3′ and reverse primer 5′- TTACAAAAATCGTAAAATCAGTCT -3′, were used for RT-PCR to determine the *TcRtv* expression profile. The *T. castaneum ribosomal protein-6* (*TcRpS6*) gene was used as an internal control for equal loading of cDNA templates [22].

### RNA interference studies

Two different regions of the *TcRtv* gene were selected for making dsRNAs. Pairs of forward and reverse primers for the chosen regions (Table S1) were used to amplify the dsRNA sequences by using the cloned *TcRtv* cDNA as template. The Ampliscribe T7-Flash Transcription Kit (Epicentre Technologies) was used to synthesize dsRNA as described previously [5]. dsRNA for the tryptophan oxygenase, *dsVermilion (dsVer)* gene, which is required for eye pigmentation, was used as a control. *dsRNAs* for *TcRtv* or *TcVer* were injected into animals in the young larval, last instar larval, or pharate pupal stages of *T. castaneum* development (200 ng per insect, n = 30). Five insects were collected at the young larval, pharate pupal and pharate adult stages of development 3–5 d after dsRNA treatment. Total RNA was extracted from the collected insects for measuring transcript levels by RT-PCR using gene-specific primer-pairs.

### Expression of recombinant TcRtv

The full-length *TcRtv* cDNA clone was used as template to amplify the complete coding region of the *TcRtv* gene. A primer pair containing appropriate restriction enzyme sites [forward primer 5′-TATCCCGGGATGGGTCTGTTTAGATC-3′ (Sma I) and a reverse primer 5′-CAGACTGATTTTACGATTTTTGTAATCTAGATAT-3′ (Xba I)] was used to facilitate directional cloning of the *TcRtv* open reading frame (ORF) DNA in the pVL1393 baculovirus expression vector (BD Pharmingen). PCR-amplified, full length *TcRtv* DNA and the pVL1393 vector DNA were digested with the same pair of restriction enzymes and ligated as described previously [15]. Hi-5 cells (*Trichoplusia ni* cell line) were used to express the TcRtv protein as described earlier [15].

### Immunohistochemistry

*TcRtv* dsRNA-treated pharate adult insects were collected and fixed as described previously [10]. Cryosections of 20 µm thickness were made and stained for specific proteins using chicken antiserum to *T. castaneum* Rtv (1∶100), rabbit antiserum (1∶100) to *D. melanogaster* Knk and rabbit antiserum to *T. castaneum* Chs-A (1∶50) as primary antibodies. Alexa 488 goat-anti-chicken IgG (1∶1000) or Alexa 488 goat-anti-rabbit IgG (1∶1000) were used as secondary antibodies for the fluorescence detection of TcRtv, TcKnk and TcChs-A proteins. Rhodamine conjugated chitin-binding probe (1∶100) and DAPI (1∶15) were used for staining of chitin and nuclei, respectively. Confocal microscopy was performed with an LSM META 510 laser scanning confocal microscope using laser lines of 405 nm, 488 nm and 543 nm for excitation. Images were taken using an oil objective (40×/1.3 NA) with 8× zoom and processed in Adobe Photoshop 7.0.

### PI-Phospholipase-C (PI-PLC) treatment

Hi-5 cells were infected with a recombinant baculovirus containing the TcRtv and TcKnk open reading frame and incubated at 30°C for 3 d for expression of proteins. Three days post-infection, Hi-5 cells expressing respective proteins were treated with PI-PLC as described previously [10].

### Chitin content analysis

Following dsRNA treatment, pharate pupae and pharate adults of *T. castaneum* were collected for chitin content analysis by a modified Morgan-Elson method as described previously [5]. *TcVer* dsRNA and *TcChs-A* dsRNA-treated insects were used as negative and positive controls for the experiment, respectively.

### Transmission electron microscopic (TEM) analysis

Pharate pupae were injected with dsRNA and pharate adult samples were collected on pupal day 5, fixed and embedded in EMBED 812/Araldite resin as described previously [10]. Resin embedded samples were then thin-sectioned (silver to gold section) and imaged on a CM-100 TEM.

### Accession numbers

cDNA sequence for *T. castaneum* Retroactive (TcRtv) is deposited at NCBI with accession number JX470185.

## Supporting Information

Figure S1 (A) Schematic diagram of the exon–intron organization of *TcRtv* gene. The exon–intron organization of *TcRtv* gene was determined by sequence comparison between genomic sequence and the full-length cDNA sequence containing 5′- and 3′-UTR regions. This gene is composed of three exons. * and closed triangle indicates start and stop codons, respectively. (B) TcRtv encodes a 15 kDa protein with an N-terminal signal peptide (red; SignalP prediction) and a C-terminal hydrophobic region (Yellow; PredGPI prediction). Ten conserved cysteine and aromatic residues are shown in blue and pink color, respectively. The ω-asparagine residue where the cleavage is predicted to occur for GPI anchoring is underlined (PredGPI).
(TIF)

Figure S2 Localization of TcChs-A in insects treated with dsRNA for *TcVer* (control, *dsTcVer*) and *TcRtv* (*dsTcRtv*). *T. castaneum* pharate adult lateral body wall sections (20 µm) were stained with TcChs-A antibody. No visible differences in the cellular distribution of TcChs-A protein localization were detected in absence of TcRtv. Chitin (red); TcChs-A (green); DAPI (blue); C, cuticle; E, epithelial cell. Scale bar = 5 µm.
(TIF)

Figure S3 TcRtv is not released to the medium by PI-PLC treatment. Recombinant TcRtv and TcKnk proteins were expressed in Hi-5 cells infected with recombinant baculoviruses containing the ORF of TcRtv or TcKnk. After 72 h of infection, the medium was removed and fresh medium was added along with 100 µl of phosphatidylinositol-specific phospholipase-C (PI-PLC) from *Bacillus cereus* (7.89 units/mg) for 4 h and the proteins in the medium and cell pellet were subjected to western blot analysis using an anti-Knk or anti-Rtv antiserum. Lanes: 1, Size marker; 2, Medium from TcRtv/TcKnk-expressing Hi-5 cells after 72 h of infection; 3, Cell pellet from TcRtv/TcKnk-expressing Hi-5 cells 72 h after infection. For lanes 4–7, old medium was removed and replaced with fresh medium with or without added PI-PLC. 4, Medium from TcRtv/TcKnk-expressing Hi5 cells after mock-treatment for 4 h without PI-PLC; 5, TcRtv/TcKnk-expressing Hi-5 cell pellet without PI-PLC treatment; 6, Medium from TcRtv/TcKnk-expressing Hi-5 cells 4 h after PI-PLC treatment; 7, Cell pellet from TcRtv/TcKnk-expressing Hi-5 cells after 4 h of PI-PLC treatment. TcRtv was found in the cell pellet fraction (lane 3 versus lane 2) and it was not released to the medium after 4 h of PI-PLC treatment (lane 6 versus lane 4). TcKnk was used as a control for PI-PLC treatment and it was released to the medium after 4 h of PI-PLC treatment (compare lane 6 versus lane 4).
(TIF)

Figure S4 Insect Rtv proteins have the conserved three-finger domain (TFD). Alignment of Rtv proteins from different insect species shows 10 conserved cysteines (solid red boxes) and aromatic residues (blue), which are predicted to bind with chitin.
(TIF)

Table S1 Primers used for dsRNA synthesis.
(DOC)
