# Supplementary figures and images for: Retroactive Maintains Cuticle Integrity by Promoting the Trafficking of Knickkopf into the Procuticle of Tribolium castaneum
Source: PLoS Genet. 2013 Jan 31;9(1):e1003268. doi: 10.1371/journal.pgen.1003268 (PMC3561106; doi:10.1371/journal.pgen.1003268)

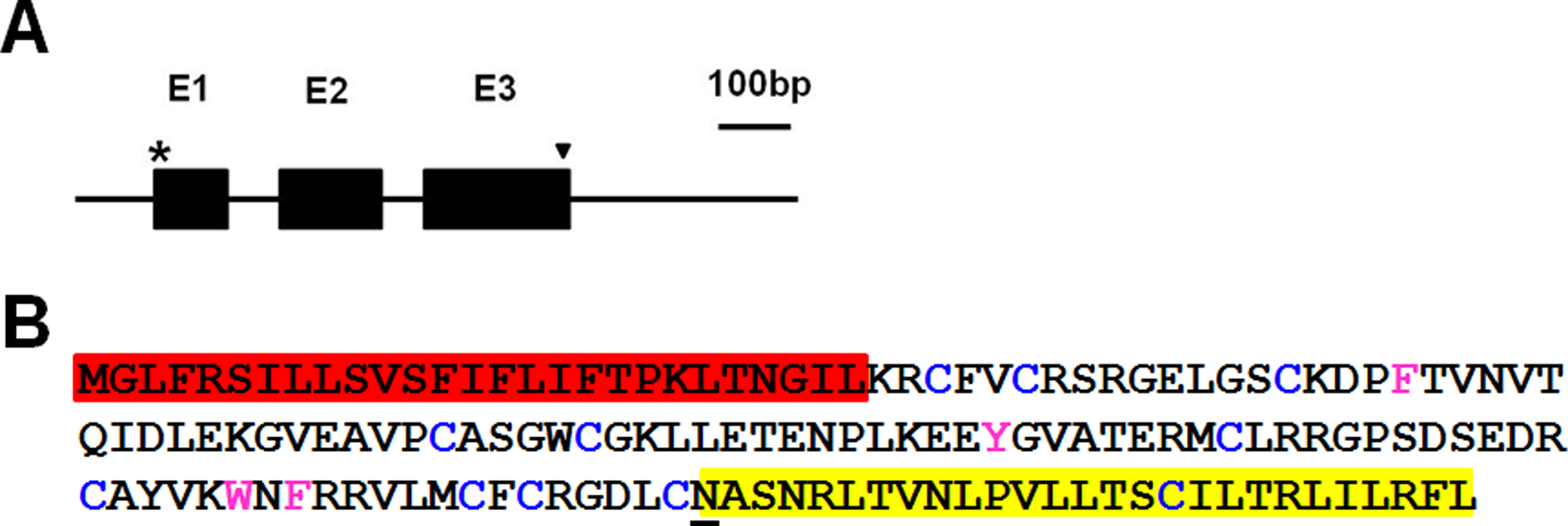

Supplement: Figure S1 — (A) Schematic diagram of the exon–intron organization of TcRtv gene. The exon–intron organization of TcRtv gene was determined by sequence comparison between genomic sequence and the full-length cDNA sequence containing 5′- and 3′-UTR regions. This gene is composed of three exons. * and closed triangle indicates start and stop codons, respectively. (B) TcRtv encodes a 15 kDa protein with an N-terminal signal peptide (red; SignalP prediction) and a C-terminal hydrophobic region (Yellow; PredGPI prediction). Ten conserved cysteine and aromatic residues are shown in blue and pink color, respectively. The ω-asparagine residue where the cleavage is predicted to occur for GPI anchoring is underlined (PredGPI). (TIF) [file pgen.1003268.s001.tif]

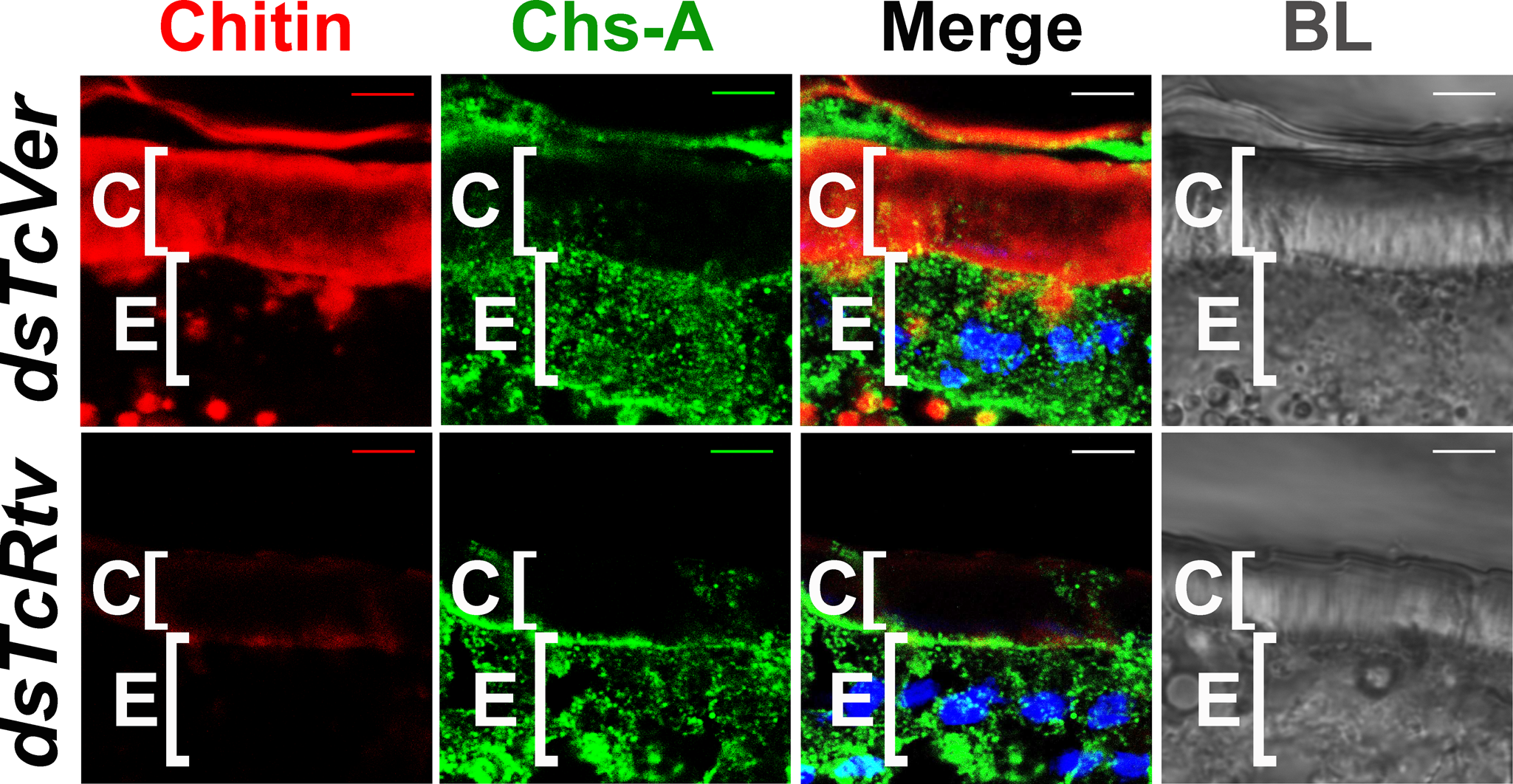

Supplement: Figure S2 — Localization of TcChs-A in insects treated with dsRNA for TcVer (control, dsTcVer) and TcRtv (dsTcRtv). T. castaneum pharate adult lateral body wall sections (20 µm) were stained with TcChs-A antibody. No visible differences in the cellular distribution of TcChs-A protein localization were detected in absence of TcRtv. Chitin (red); TcChs-A (green); DAPI (blue); C, cuticle; E, epithelial cell. Scale bar = 5 µm. (TIF) [file pgen.1003268.s002.tif]

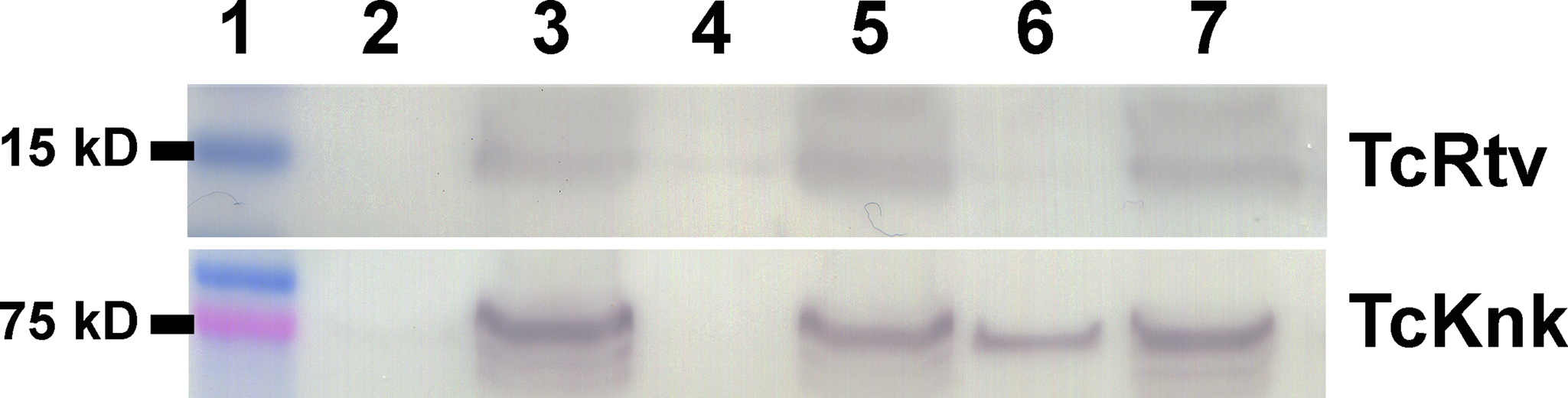

Supplement: Figure S3 — TcRtv is not released to the medium by PI-PLC treatment. Recombinant TcRtv and TcKnk proteins were expressed in Hi-5 cells infected with recombinant baculoviruses containing the ORF of TcRtv or TcKnk. After 72 h of infection, the medium was removed and fresh medium was added along with 100 µl of phosphatidylinositol-specific phospholipase-C (PI-PLC) from Bacillus cereus (7.89 units/mg) for 4 h and the proteins in the medium and cell pellet were subjected to western blot analysis using an anti-Knk or anti-Rtv antiserum. Lanes: 1, Size marker; 2, Medium from TcRtv/TcKnk-expressing Hi-5 cells after 72 h of infection; 3, Cell pellet from TcRtv/TcKnk-expressing Hi-5 cells 72 h after infection. For lanes 4–7, old medium was removed and replaced with fresh medium with or without added PI-PLC. 4, Medium from TcRtv/TcKnk-expressing Hi5 cells after mock-treatment for 4 h without PI-PLC; 5, TcRtv/TcKnk-expressing Hi-5 cell pellet without PI-PLC treatment; 6, Medium from TcRtv/TcKnk-expressing Hi-5 cells 4 h after PI-PLC treatment; 7, Cell pellet from TcRtv/TcKnk-expressing Hi-5 cells after 4 h of PI-PLC treatment. TcRtv was found in the cell pellet fraction (lane 3 versus lane 2) and it was not released to the medium after 4 h of PI-PLC treatment (lane 6 versus lane 4). TcKnk was used as a control for PI-PLC treatment and it was released to the medium after 4 h of PI-PLC treatment (compare lane 6 versus lane 4). (TIF) [file pgen.1003268.s003.tif]

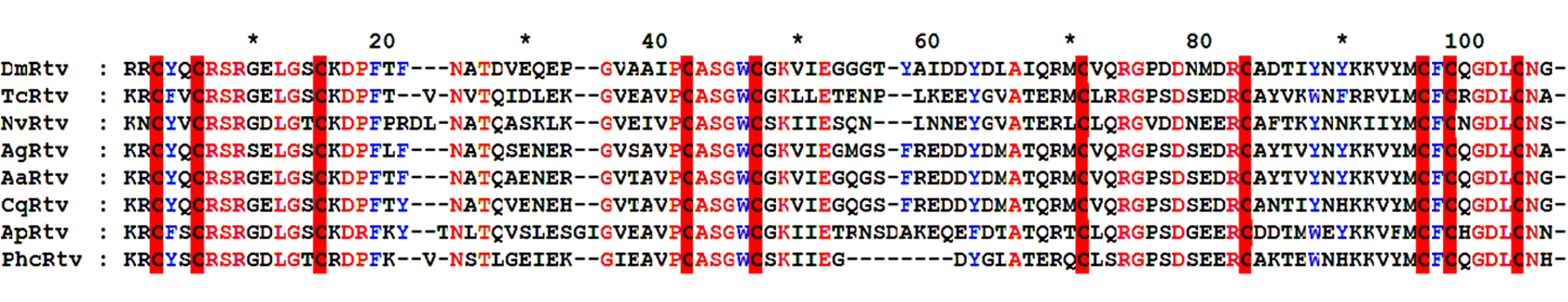

Supplement: Figure S4 — Insect Rtv proteins have the conserved three-finger domain (TFD). Alignment of Rtv proteins from different insect species shows 10 conserved cysteines (solid red boxes) and aromatic residues (blue), which are predicted to bind with chitin. (TIF) [file pgen.1003268.s004.tif]
