# Supplementary material for: Retroactive Maintains Cuticle Integrity by Promoting the Trafficking of Knickkopf into the Procuticle of Tribolium castaneum
Source: PLoS Genet. 2013 Jan 31;9(1):e1003268. doi: 10.1371/journal.pgen.1003268 (PMC3561106; doi:10.1371/journal.pgen.1003268)
Supplement: Table S1 — Primers used for dsRNA synthesis. (DOC) [file pgen.1003268.s005.doc]

**Table S1. Primers used for dsRNA synthesis.**

| dsRNA |  | Sequence (5’-3’) | Nucleotide positions | dsRNA Length, bp |
| --- | --- | --- | --- | --- |
| 1 | F= | TAATACGACTCACTATAGGGTGGAATTCTCAAACGC | 72-233 | 161 |
|  | R= | TAATACGACTCACTATAGGGTGGATTTTCTGTCTCT |  |  |
| 2 | F= | TAATACGACTCACTATAGGGTTTGTATGCCGATCTC | 91-365 | 274 |
|  | R= | TAATACGACTCACTATAGGGTAAGTCTCCTCGGCAG |  |  |
